# Self-Powered Ultraviolet Photodetectors Based on Conductive Polymers/Ga_2_O_3_ Heterojunctions: A Review

**DOI:** 10.3390/polym17101384

**Published:** 2025-05-17

**Authors:** Zerui Xiao, Haoyan Chen, Honglong Ning, Dongxiang Luo, Xuecong Fang, Muyun Li, Guoping Su, Han He, Rihui Yao, Junbiao Peng

**Affiliations:** 1 State Key Laboratory of Luminescent Materials and Devices, Guangdong Basic Research Center of Excellence for Energy and Information Polymer Materials, South China University of Technology, Guangzhou 510640, China; 202230274330@mail.scut.edu.cn (Z.X.); 202420119664@mail.scut.edu.cn (H.C.); 202421021983@mail.scut.edu.cn (X.F.); 202111084406@mail.edu.scut.cn (M.L.); 201730321254@mail.scut.edu.cn (G.S.); 202410184224@mail.scut.edu.cn (H.H.); psjbpeng@scut.edu.cn (J.P.); 2 The International School of Microelectronics, Dongguan University of Technology, Dongguan 523808, China; 3 Huangpu Hydrogen Innovation Center/Guangzhou Key Laboratory for Clean Energy and Materials, School of Chemistry and Chemical Engineering, Guangzhou University, Guangzhou 510006, China; luodx@gzhu.edu.cn

**Keywords:** solar-blind UV photodetector, gallium oxide, conductive polymer, self-powered, polythiophene, polyaniline, wide bandgap

## Abstract

Self-powered ultraviolet photodetectors hold significant potential for diverse applications across both military and civilian fields. Owing to its wide bandgap, high electron mobility, and adaptability to various substrates, gallium oxide (Ga_2_O_3_) serves as a crucial material for fabricating self-powered ultraviolet photodetectors. Photodetectors based on p-n heterojunctions of conductive polymers and gallium oxide have great application potential benefiting from unique advantages of conductive polymers. This review provides an extensive overview of typical ultraviolet photodetectors based on conductive polymer/gallium oxide heterojunctions, focusing on the physical structure, fabrication process, and photoelectric properties of heterojunction devices formed by Ga_2_O_3_ with conductive polymers like polythiophene, polyaniline, and polycarbazole, etc. Different conductive polymers yield varying performance improvements in the fabricated devices: polythiophene/Ga_2_O_3_ devices exhibit high conductivity and flexible bandgap tuning to meet diverse wavelength detection needs; PANI/Ga_2_O_3_ devices feature simple fabrication and low cost, with doping control to enhance charge carrier transport efficiency; polycarbazole/Ga_2_O_3_ devices offer high thermal stability and efficient hole transport. Among them, the polythiophene/Ga_2_O_3_ device demonstrates the most superior overall performance, making it the ideal choice for high-performance Ga_2_O_3_-based photodetectors and a representative of such research. This review identifies the existing technical challenges and provides valuable insights for designing more efficient Ga_2_O_3_/conductive polymer heterojunction photodetectors.

## 1. Introduction

Ultraviolet (UV) radiation, a vital segment of the electromagnetic spectrum ranging from 10 to 400 nm and situated between visible light and X-rays, is indispensable to human technological progress and natural life processes [1]. Depending on the wavelength, UV light can be further categorized into EUV (10–120 nm), UVC (100–280 nm), UVB (280–320 nm), and UVA (320–400 nm) [2]. Among these, UV radiation with wavelengths between 10 and 280 nm has garnered extensive attention due to its unique “zero background noise” characteristic. This specific UV band is almost entirely absorbed by atmospheric ozone molecules, resulting in a solar radiation intensity at the Earth’s surface of less than 10^−14^ W/cm^2^, thus forming a natural “optical darkroom”. Consequently, it is also referred to as the solar-blind UV band [3]. The solar-blind UV band enables extremely low-noise detection without the need for optical filters and holds significant application value in both civilian and military fields such as space secure communication, fire monitoring, and missile early warning [4,5].

According to photovoltaic theory, wide-bandgap semiconductor materials have strong application potential in the detection of the solar-blind UV spectrum and have attracted great attention from researchers in recent years. Wide-bandgap semiconductor materials such as gallium oxide (Ga_2_O_3_), tin oxide (SnO), zinc oxide (ZnO), aluminum nitride (AlN), gallium nitride (GaN), and diamond [6,7,8,9,10,11] have good spectral selectivity at room temperature due to their wide bandgaps, and they also have high material stability and good thermal conductivity, making them ideal material choices for the fabrication of efficient solar-blind UV photodetectors. In particular, gallium oxide (Ga_2_O_3_) has become a frontier material in the research of solar-blind UV photodetectors in recent years due to its characteristics of a wide bandgap (4.2~5.3 eV), high breakdown voltage (8 MV cm^−1^), and high electron mobility (200–300 cm^2^·V^−1^·s^−1^), as well as lower fabrication temperatures, more flexible substrate selection, and better uniformity [12,13].

Gallium oxide can be fabricated into various forms of UV photodetectors, including bulk materials, thin films, nanorods, and others, and it can also be combined with other doped materials to form hybrid heterojunction detectors. Depending on their structure and principles, common Ga_2_O_3_ UV PDs are mainly divided into several types, including photoconductive, Schottky-type, p-n (p-i-n) junction-type, metal-semiconductor-metal (MSM)-type, avalanche photodiodes (APDs), and phototransistors [14,15,16,17,18,19]. Among these, the p-n junction type is often used to build self-powered photodetectors because it can form a space charge region, generate a built-in electric field, and operate without external bias [20]. However, as p-type doping of Ga_2_O_3_ has not been achieved, it is difficult to construct homogeneous p-n or p-i-n junction UV photodetectors that can work in photovoltaic mode [21]. Therefore, it is common to choose p-type semiconductors or n-type semiconductors with large band offsets to construct p-n or n-n Ga_2_O_3_ heterojunctions as an alternative method. There have been numerous attempts to integrate n-type Ga_2_O_3_ with inorganic p-type materials into heterojunctions, such as NiO/Ga_2_O_3_, Cu_2_O/α-Ga_2_O_3_, CuI/β-Ga_2_O_3_, SnO_2_/β-Ga_2_O_3_, and p-GaN/β-Ga_2_O_3_ [22,23,24,25,26]. However, the construction of inorganic heterojunctions faces some challenges. Firstly, inorganic materials often require fabrication under high-temperature and high-vacuum conditions, and some involve a large number of lithography steps, making the manufacturing and processing processes difficult. In addition, due to the different lattice constants of different inorganic materials, lattice mismatch often occurs at the interface of epitaxial heterojunctions, leading to the generation of interface states that capture charge carriers, reducing the separation and collection efficiency of photogenerated charge carriers, and thus limiting the external quantum efficiency and suppression ratio of the devices [27].

In recent years, conductive polymers (CPs) have gained more attention from researchers due to their unique advantages, offering new ideas for the manufacture of more economical and efficient self-powered gallium oxide detectors. Compared with inorganic materials, organic materials can be prepared at lower temperatures using simpler methods [28,29,30]. In addition, some organic materials are not sensitive to surface defects, and their interface interaction with Ga_2_O_3_ is much weaker than that of inorganic materials. This improves the lattice mismatch phenomenon and greatly enhances the external quantum efficiency of the device. Unlike small molecule organics, conductive polymers conduct electricity through the conjugated π—electrons in the backbone. One of their major features is that the conductivity can be adjusted over several orders of magnitude by regulating the doping level. Therefore, choosing a properly doped p-type organic semiconductor can result in a pn heterojunction with a high hole transport rate [31,32]. Organic−inorganic hybrid heterojunction devices constructed using conductive polymers can promote carrier separation, significantly improve photoelectric performance, and also reduce manufacturing costs and increase device flexibility. In recent years, they have been favored by an increasing number of researchers [33].

**Figure 1:**
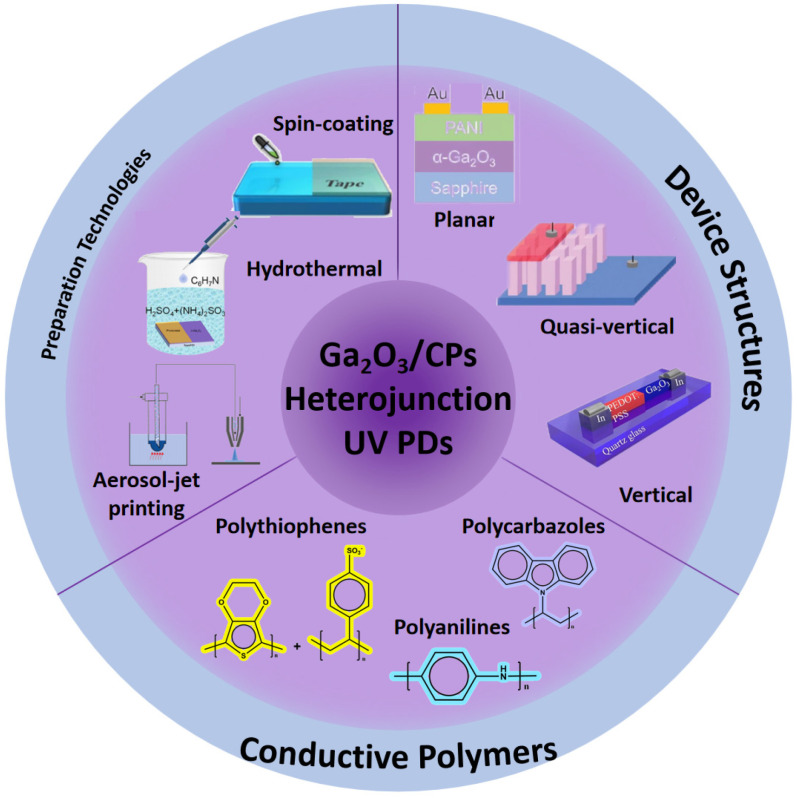
Outline of conductive polymers/Ga_2_O_3_ self-powered ultraviolet photodetectors, reproduced from [34].

This paper reviews the recent progress in UV photodetectors based on Ga_2_O_3_ and conductive polymer heterojunctions, as illustrated in Figure 1. After introducing the structure and performance parameters of Ga_2_O_3_/CPs UV PDs, it delves into the structure, properties of Ga_2_O_3_ and conductive polymers, and heterojunction fabrication methods to clarify the contribution of conductive polymers to Ga_2_O_3_ photodetectors. Then, it examines key developments in self-powered UV photodetectors with Ga_2_O_3_/CPs heterojunctions. Finally, it summarizes the current issues and prospects of Ga_2_O_3_/CPs UV PDs.

## 2. Structures and Parameters of Ga_2_O_3_/CPs UV PDs

### 2.1. Structures of Ga_2_O_3_/CPs UV PDs

When n-type doped gallium oxide and p-type doped conductive polymers are arranged into a p-n junction, charge diffusion causes the Fermi levels of the p and n regions to align, forming a depletion region and creating a built-in electric field that balances the diffusion process [35]. When UV light illuminates the p-n junction of the detector, high-energy photons with energy exceeding the bandgap are absorbed by the semiconductor material, generating charge carriers. Under the action of the electric field at the p-n junction, the electron–hole pairs are rapidly separated and move directionally to form a current. This photodiode can operate at zero bias or under reverse bias. At zero bias, the relatively low dark current can enhance the device’s specific detectivity (D*) and sensitivity. Under reverse bias, the expanded depletion region shortens the carrier transport time and diode capacitance, thus improving the response speed. As a variant of p-n junctions, p-i-n photodiodes have an added transparent intrinsic layer between the p+ and n+ contact layers, which is beneficial for light absorption. This structure allows for precise control of the depletion region thickness, reduces carrier diffusion between different doping regions, and optimizes quantum efficiency and response speed, making it highly regarded. Compared to other Ga_2_O_3_-based devices, the fabrication of p-n (p-i-n) photodiodes is more complex, as it requires the deposition of semiconductor thin films with different doping types to form the p-n (p-i-n) junction. Although Ga_2_O_3_ cannot form p-n homojunctions due to ineffective p-type doping, p-n heterojunctions can be formed with other p-type semiconductors. Among these, p-type conductive polymers offer significant potential for self-powered detectors due to their lower cost and higher photoelectric performance, and they have been the subject of extensive research in recent years.

**Figure 2:**
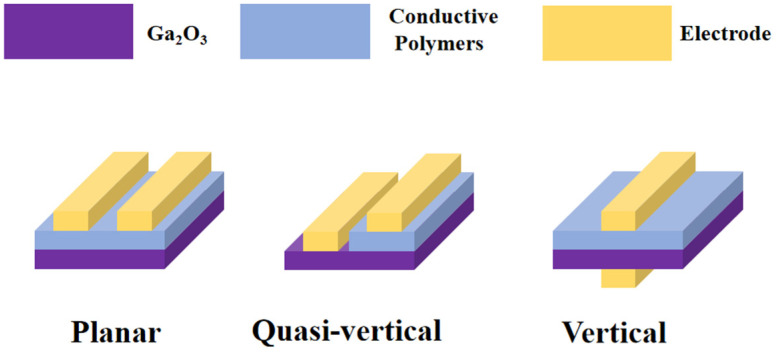
Schematic diagram of geometric configurations of Ga_2_O_3_/CPs heterojunction UV PDs.

Depending on the arrangement of the materials and electrodes, Ga_2_O_3_/CPs heterojunction UV PDs are typically categorized into several geometric configurations: planar, quasi-vertical, and vertical, as illustrated in Figure 2.

The planar structure represents the most fundamental geometric configuration, where two electrodes are arranged in parallel on the same side of the gallium oxide layer [36]. This setup is simple to construct and cost−effective, requiring only straightforward deposition and photolithography to form the metal contact patterns during fabrication. However, the lateral collection efficiency of carriers is restricted, leading to a moderate response time of the device. Moreover, the thick photoactive layer in such devices limits the light absorption path, resulting in a poor response rate. Planar structure devices are suitable for applications with low fabrication requirements and modest performance demands regarding sensitivity, response rate, etc., such as flexible photodetectors and photosensors.

The quasi-vertical structure enhances the planar structure by adjusting the angle between the electrodes and the photoactive layer, thereby reducing the carrier transport path [37]. This allows light to penetrate deeper into the active layer, reducing lateral carrier transport losses and increasing the response speed compared to the planar structure. However, the fabrication process for quasi-vertical devices is more complex and costly.

Vertical structure devices have electrodes arranged on opposite faces of the gallium oxide, extending the light path and enhancing light absorption, with improved carrier collection efficiency [38]. Light is incident vertically on the device, directly exciting carriers in the active layer, which then move vertically towards the electrodes under the electric field, shortening the carrier transport path and significantly increasing the response speed. Vertical structure devices often utilize nanorod-shaped gallium oxide for preparation. This structure requires a uniformly thick active layer and precise interface treatment, with a complex fabrication process and high cost.

In summary, vertical devices perform the best in terms of response speed, quasi-vertical devices come next, and planar devices are third. However, planar devices are the most cost-effective, vertical devices are the most expensive, and quasi-vertical devices lie in between. Thus, the choice of device configuration should be based on actual requirements.

### 2.2. Parameters of Ga_2_O_3_/CPs UV PDs

Ultraviolet photodetectors involve the conversion of light signals into electrical signals. The main parameters commonly used to evaluate the photoelectric performance of photodetectors are as follows [39,40,41,42,43].

#### 2.2.1. Responsivity

The responsivity of a photodetector is defined as the ratio of the output electrical signal (current or voltage) to the incident light power, expressed per unit area. It is commonly used to assess the sensitivity of the photodetector device and can be calculated using Equation (1).

(1) $$R=\frac{I}{P\cdot S}$$

Herein, R is the responsivity, I is defined as the current generated by the photodetector, while P represents the incident light power, and S refers to the effective area of the photodetector.

#### 2.2.2. Rejection Ratio

The rejection ratio refers to the ratio of the responsivity of a detector to specific wavelength ultraviolet light compared to visible light. It is used to measure the selectivity of solar-blind ultraviolet detectors. Typically, the responsivity of the detector at a specific ultraviolet wavelength (such as 254 nm) and a visible light wavelength (such as 600 nm) is measured, and then the ratio of the two is calculated. For example, the rejection ratio at 254 nm and 600 nm can be expressed as R_254nm_/R_600nm_.

#### 2.2.3. Detectivity

Detectivity is a parameter that measures the performance of a detector. It is related to the signal-to-noise ratio and the noise equivalent power of the detector. It can be calculated using Equation (2).

(2) $$D^{*}=\frac{R}{\sqrt{2qI_{d}+I^{2}}}$$

Herein, D* is the detectivity, R is defined as the responsivity, q refers to the electron charge, while I_d_ represents the dark current, and I stands for the photocurrent.

#### 2.2.4. External Quantum Efficiency (EQE)

Based on the photoelectric effect, the energy of absorbed photons is converted into the kinetic energy of ejected electrons. However, not all incident light is absorbed by the device due to effects like reflection. The ratio of the number of generated electrons to the total number of incident photons is known as the External Quantum Efficiency (EQE). EQE characterizes the device’s efficiency in converting photons to electrons and can be calculated using Equation (3).

(3) $$\eta_{\mathrm{EQE}}=R\frac{h\nu }{q}$$

Herein, $\eta_{\mathrm{EQE}}$ is the external quantum efficiency, R is defined as the responsivity, q refers to the electron charge, while h represents the Planck’s constant, and ν is the frequency of the incident light. Generally, the higher the EQE of a UV PD, the more photocarriers are generated per photon within the UV PD.

#### 2.2.5. Response Time

Response time includes both rise time and fall time, referring to the duration it takes for the detector to transition from a state of no light to light, or from light to no light. The transport of photogenerated carriers from the semiconductor material to the electrodes requires a certain amount of time. By measuring the changes in current or voltage of the detector when the light signal changes, the rise time is defined as the time it takes for the current or voltage to increase from 10% to 90%, while the fall time is defined as the time required for it to decrease from 90% to 10%. Improving the structural properties of the photoanode material allows for the quick separation and rapid transport of electron–hole pairs, thereby enhancing the response time.

#### 2.2.6. Open-Circuit Voltage, Self-Powered Current, and Dark Current

The open-circuit voltage (V_OC_) refers to the voltage at the terminals of the detector when there is no external load, which can be measured by applying zero bias to the detector. The self-powered current refers to the current generated by the photovoltaic effect without external power supply, which can be measured by measuring the current at the terminals of the detector under illumination. The dark current (I_dark_) refers to the current signal measured in the dark state of the device, which is the main source of interference for UV detectors. For a given bias voltage, a lower dark current indicates better performance of the UV PD. The suppression of dark current usually depends largely on the preamplifier circuit of the system.

## 3. Structures and Properties of Ga_2_O_3_ and Conductive Polymers in Ga_2_O_3_/CPs UV PDs

### 3.1. Structures and Properties of Gallium Oxide in Ga_2_O_3_-Based UV PDs

**Figure 3:**
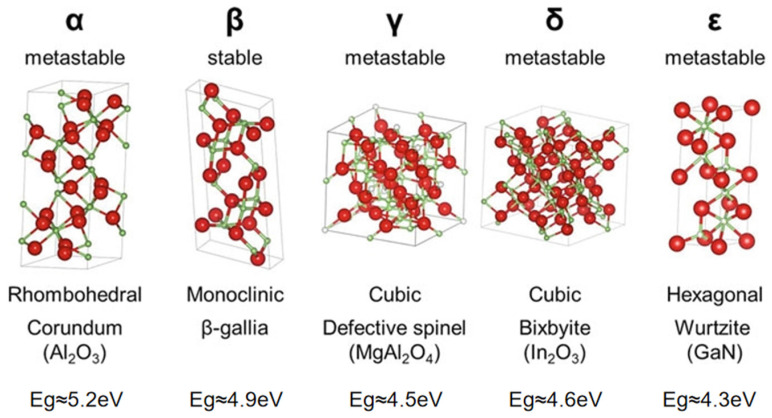
Schematic diagram of various crystal structures and bandgap widths of gallium oxide (Ga_2_O_3_), reproduced with permission from Higashiwaki, M., Fujita, S. [45], Gallium Oxide: Materials Properties, Crystal Growth, and Devices; published by Springer (Berlin/Heidelberg, Germany), 2020.

There are five well-known crystallographic phases of gallium oxide, namely α-Ga_2_O_3_, β-Ga_2_O_3_, γ-Ga_2_O_3_, δ-Ga_2_O_3_, and ε-Ga_2_O_3_. The structural diagrams of each phase and their corresponding bandgap widths are shown in Figure 3 [44,45]. In addition, a transient phase known as κ-Ga_2_O_3_ has also garnered increasing attention in recent years. Its crystal structure is very similar to that of hexagonal ε-Ga_2_O_3_, and it is expected to exhibit similar polarization properties [46].

β-Ga_2_O_3_ is the most stable phase, which belongs to the C2/m space group with lattice constants of a = 12.2 Å, b = 3.0 Å, and c = 5.8 Å. It has a monoclinic structure, with an angle of approximately 104° between the a-axis and the c–axis [47,48]. Compared with third-generation semiconductors such as SiC and GaN, β-Ga_2_O_3_ has a larger bandgap, a shorter absorption cutoff edge, and lower growth costs. It does not require alloying processes like those for AlGaN and ZnMgO, making it an ideal material for the fabrication of solar-blind UV detectors. The bandgap of β-Ga_2_O_3_ is about 4.9 eV, corresponding to a cutoff wavelength of approximately 260 nm, which just covers the solar-blind band. This eliminates the need to adjust the bandgap during the fabrication process. Moreover, even at the band edge, β-Ga_2_O_3_ has an absorption coefficient greater than 10^5^ cm^−1^, resulting in a high rejection ratio for gallium oxide detectors [49,50,51]. The Baliga figure of merit (εμE_g_^3^, relative to Si) of β-Ga_2_O_3_ is as high as 3214.1, which is about 10 times that of SiC and 4 times that of GaN. This implies that devices fabricated using β-Ga_2_O_3_ will have lower conduction losses and higher power conversion efficiency, making it suitable for high-voltage and high-power devices [52,53,54].

α-Ga_2_O_3_ is the second most studied polymorph and is representative of the four metastable phases (α -, γ -, δ -, and ε-Ga_2_O_3_) [55]. α-Ga_2_O_3_ thin films are typically formed on sapphire substrates through low-temperature heteroepitaxial growth [56]. The bandgap width of α-Ga_2_O_3_ is estimated to be about 5.2 eV based on optical absorption measurements. In fact, the heteroepitaxial growth method can also be used to prepare metastable phases of gallium oxide such as γ-Ga_2_O_3_ and ε-Ga_2_O_3_ [57].

Controlling the heating temperature and humidity can yield different Ga_2_O_3_ polymorphs, which are also mutually convertible. Rapidly heating Gallia Gels to 400–500 °C produces γ-Ga_2_O_3_, which further converts to α-Ga_2_O_3_ with prolonged heating. δ-Ga_2_O_3_ is obtained by heating gallium nitrate at 250 °C, and continued heating under humid conditions above 300 °C transforms it into α-Ga_2_O_3_, while heating under dry conditions above 500 °C yields ε-Ga_2_O_3_, also obtainable by drying Ga_2-x_Al_x_O_3_ [47]. All polymorphs convert to β-Ga_2_O_3_ upon heating above 870 °C or under hydrothermal conditions above 300 °C. The transient κ-Ga_2_O_3_ phase appears during intermediate stages of phase transitions, of which the formation is highly sensitive to reaction parameters such as temperature, time, and atmosphere. This transient phase often serves as a precursor or intermediate step in the synthesis of more stable Ga_2_O_3_ polymorphs and can be transformed into phases such as α-Ga_2_O_3_ or β-Ga_2_O_3_ through subsequent thermal or chemical treatments.

### 3.2. Structures and Properties of Conductive Polymers in Ga_2_O_3_/CPs UV PDs

Polymers are large molecules formed by the polymerization process, where many repeating small units are linked together by covalent bonds. These repeating small units are called monomers. Due to the highly bound electrons in their structure, which do not facilitate electron flow, polymers have a high resistivity and have long been widely used as insulators in electrical and electronic applications [58]. It was not until 1977 that Chiang and Shirakawa et al. [59] discovered that polyacetylene doped with iodine could exhibit conductivity comparable to that of metals. This finding shocked the academic community, was epoch-making, and opened up a new research field. Subsequent research has found that if the electrons in a polymer can move as freely as those in metals, the polymer can become a conductive “metal,” known as a conductive polymer (CP). The key to the free movement of electrons throughout the conjugated system is the conjugated system and doping. In a conjugated system, electrons can “delocalize,” meaning they can move in the molecular orbitals formed by the integration of several adjacent atoms, rather than being confined to their original atomic orbitals. Dopants can enhance the conductivity of the polymer by generating additional electrons (through reduction) or holes (through oxidation). The absence of an electron creates a hole, which is then filled by an electron jumping from a neighboring position, thus forming a new hole and enabling charge to migrate over long distances [60].

Common methods for synthesizing CPs include chemical preparation and electrochemical preparation [61]. Compared to chemical preparation, electrochemical preparation is cleaner and more environmentally friendly, and the physical form of the product is also easier to control. However, electrochemical preparation also has some drawbacks, such as the product’s geometric form and properties being entirely dependent on the substrate, and the loss of redox properties after electrode deposition [62,63].

**Figure 4:**
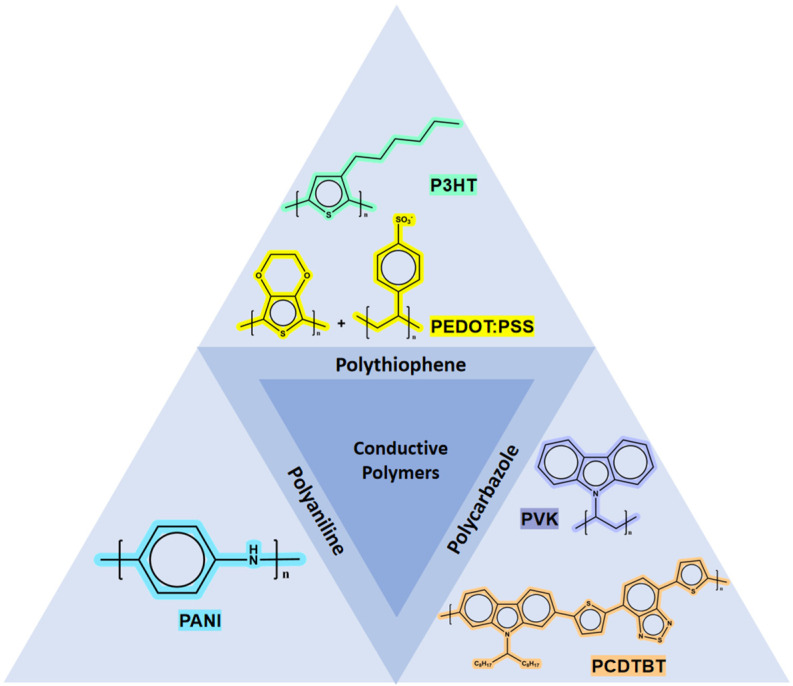
Schematic diagram of structures of commonly used conductive polymers for Ga_2_O_3_ photodetectors.

Following the discovery of polyacetylene, researchers continued to explore numerous potential conducting polymers, achieving many remarkable results. One significant characteristic of CPs is their broad range of conductivity. Electrons and holes can form and move within the conjugated backbone composed of alternating single and double bonds and can be further regulated according to different doping levels. Compared to inorganic semiconductors and metals, CPs offer more pronounced advantages, making them highly promising as multifunctional materials for applications in chemical sensors, optical devices, and biomedical devices [64]. Gallium oxide can form hybrid organic-inorganic p-n heterojunctions with p-type doped conducting polymers. Solar-blind UV photodetectors based on this structure exhibit faster hole mobility and superior visible light absorption performance, which has also attracted significant attention. There have been reports on the fabrication of mixed heterojunctions using Ga_2_O_3_ with various types of conducting polymers, such as polythiophenes (PEDOT:PSS, P3HT), polycarbazoles (PCDTBT, PVK), and polyanilines (PANI). Figure 4 illustrates the structural diagrams of these conducting polymers.

## 4. Preparation Methods of Ga_2_O_3_/CPs Heterojunctions

Studies have shown that photodetectors made of Ga_2_O_3_ thin films or nanostructured Ga_2_O_3_ exhibit excellent photoelectric detection performance. The most common methods for growing Ga_2_O_3_ thin films are molecular beam epitaxy (MBE) on sapphire substrates, metal–organic chemical vapor deposition (MOCVD), and radio frequency magnetron sputtering (RFMS) [65,66,67]. Other methods include sol–gel [68], pulsed laser deposition (PLD) [69], mist-CVD [66], and atomic layer deposition (ALD) [70]. For nanostructured Ga_2_O_3_, researchers have employed various synthesis methods, such as chemical vapor deposition [71], direct evaporation [72], laser molecular beam epitaxy [73], mechanical exfoliation [74], and hydrothermal synthesis [75]. Different forms of Ga_2_O_3_ are suitable for constructing devices with different geometries. Ga_2_O_3_ thin films are commonly used to build planar and quasi-vertical devices, while vertical devices are typically fabricated using Ga_2_O_3_ nanowires. Considering both the photoelectric performance and the fabrication cost, quasi-vertical devices constructed with Ga_2_O_3_ films are widely used in Ga_2_O_3_/CPs SB UV PDs. Therefore, the comparison is made among devices constructed with Ga_2_O_3_ films, when this review discusses photoelectric performances of devices and demonstrates the improvement of conductive polymers on device performances, thereby excluding the influence of the nanostructure of Ga_2_O_3_ on device performances.

**Figure 5:**
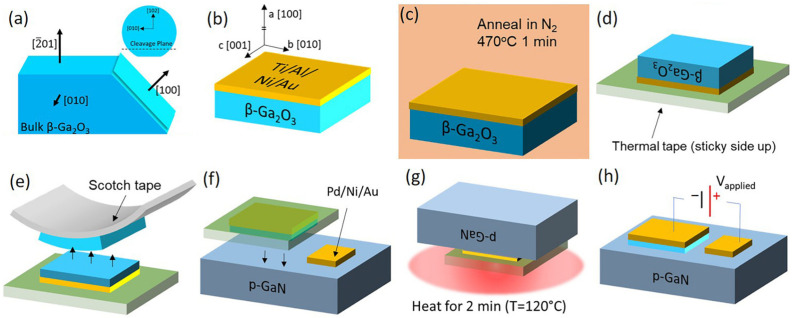
Mechanical exfoliation of β-Ga_2_O_3_. (**a**) The bulk {201} wafers of β-Ga_2_O_3_ can be cleaved to expose the {100} plane. (**b**) Metal deposition via electron beam evaporation to deposit the n contact on β-Ga_2_O_3_. (**c**) Anneal the contact in highpurity N_2_ at 470 °C for 1 min. (**d**) Thermal tape is placed over the β-Ga_2_O_3_, metal stack and turned upside down. (**e**) Ordinary scotch tape is placed sticky side-down over the exposed β-Ga_2_O_3_ and peeled off, removing layers of the β-Ga_2_O_3_. (**f**) The β-Ga_2_O_3_, metal, thermal tape is placed on p-type GaN, which had a p-contact deposited beforehand. (**g**) The entire stack is placed upside down on a vacuum-sealed hot plate at 120 °C for 2 min to evenly distribute the heat across the thermal tape. (**h**) The finished device, reproduced with permission from Montes J. et al. [79], demonstration of mechanically exfoliated β-Ga_2_O_3_/GaN p-n heterojunction; published by AIP Publishing (Melville, NY, USA), 2019.

Due to its unique property of having a large lattice constant along the [76] direction, it is easy to cut β-Ga_2_O_3_ into nanomembranes or thin strips [77]. In addition, the monoclinic crystal form of β-Ga_2_O_3_ has significant crystal anisotropy. It is not easy to cleave between different crystal planes. However, the binding force between the (100) planes and the (001) planes is weak, making cleavage easy. This makes it possible to mechanically exfoliate gallium oxide micropieces. Moreover, this method can avoid the crystal cracking problems caused by traditional grinding and polishing methods. Therefore, some research groups have attempted to use the mechanical exfoliation method to prepare gallium oxide thin-film structures [78]. Figure 5 shows an example of fabricating β-Ga_2_O_3_/GaN devices using mechanically exfoliated β-Ga_2_O_3_ films.

**Figure 6:**
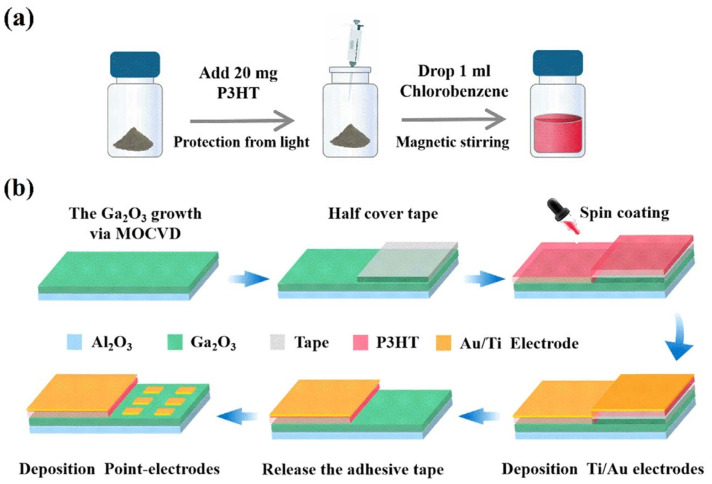
Preparation process of (**a**) the P3HT precursor solution and (**b**) the hybrid organic-inorganic P3HT/Ga_2_O_3_ p-n heterojunction device, reproduced with permission from Qi, X. et al. [81], a self-powered deep-ultraviolet photodetector based on a hybrid organic-inorganic p-P3HT/n-Ga_2_O_3_ heterostructure; published by IOP Publishing Ltd. (Bristol, UK), 2022.

Conductive polymers can be added via spin-coating, hydrothermal methods, and aerosol-jet printing, with spin-coating being the most widely used. This involves spin-coating a solution of conductive polymers onto Ga_2_O_3_ thin films or nanorods prepared by methods such as MOCVD or hydrothermal synthesis [80]. For instance, Fan et al. [75] spin-coated an aqueous solution of PEDOT:PSS (1.5% by mass) onto nanorod arrays of hydrothermally synthesized α-Ga_2_O_3_/FTO, followed by annealing, to fabricate a self-powered PEDOT:PSS/α-Ga_2_O_3_ nanorod array/FTO photodetector capable of dual-band detection of solar-blind and visible spectra. Figure 6 illustrates the process of preparing a mixed heterojunction photodiode by spin-coating a P3HT solution onto a MOCVD-prepared Ga_2_O_3_ thin film [81]. The spin-coating wet process for preparing conductive polymer thin films can minimize surface damage, prevent lattice mismatch, and form better Schottky junctions.

## 5. Types of Self-Powered Polymer-Gallium Oxide Ultraviolet Photodetectors

The self-powered solar-blind ultraviolet photodetectors based on conductive polymer/Ga_2_O_3_ heterojunction can form a built-in electric field at the junction. This allows for the rapid and effective separation and transport of electron–hole pairs without external driving forces (self-powered), demonstrating faster photoconductive response. Moreover, the compact size of self-powered detectors makes them advantageous for integrated manufacturing. To date, various Ga_2_O_3_/conductive polymer-based photodetectors have been reported. Among them, heterojunction devices fabricated from polythiophene, polyaniline, and polycarbazole derivatives have shown significant performance improvements and have been more extensively studied.

### 5.1. Types of Polythiophenes/Ga_2_O_3_-Based UV PDs

Poly (3,4-ethylenedioxythiophene):poly (styrene sulfonate) (PEDOT:PSS) is a typical conducting polymer of the polythiophene family. It is a p-doped semiconductor where the sulfonate anions in the PSS chains compensate for the holes in the PEDOT chains. Electrons are transported in the PEDOT-rich regions, while cations are transported in the PSS-rich regions, making it an ion–electron hybrid conductor with relatively high conductivity [82]. PEDOT:PSS is water-soluble, mostly semi-transparent, easy to deposit, and stable in nature [83]. It has excellent optoelectronic properties, tunable work function, high thermoelectric properties, good mechanical properties, and high thermal stability, making it one of the most promising conductive polymers. By changing the type of dopant and the oxidation state, the electrical conductivity of PEDOT:PSS can be adjusted over several orders of magnitude, making it a suitable choice for building optoelectronic detectors as an excellent hole-transporting layer. Among various types of Ga_2_O_3_ and conductive polymer heterojunctions, organic–inorganic hybrid p-n junctions constructed using PEDOT:PSS and Ga_2_O_3_ are the most extensively studied.

**Figure 7:**
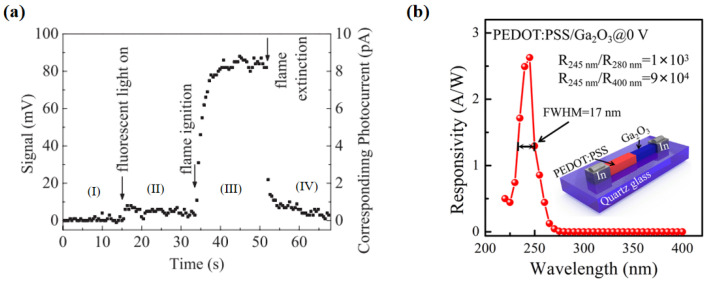
(**a**) Signals emitted by the flame detection system during the demonstration, with the experimental conditions in sequence being (I) darkness, (II) fluorescent light illumination, (III) lighter ignition, and (IV) lighter extinguishment, reproduced with permission from Oshima, T. et al. [84], Flame Detection by a β-Ga_2_O_3_-Based Sensor; published by IOP Publishing Ltd., 2009. (**b**) Structure of the PEDOT:PSS/β-Ga_2_O_3_ heterojunction detector and the photoresponse spectrum of the device at 0 V, as well as the rejection ratio, reproduced with permission from Wang, H. et al. [86], High Responsivity and High Rejection Ratio of Self-Powered SolarBlind Ultraviolet Photodetector Based on PEDOT:PSS/β-Ga_2_O_3_ Organic/Inorganic p−n Junction; published by AMER CHEMICAL SOC (Washington, DC, USA), 2019.

Early research on these detectors was performed in 2009 by Oshima et al. [84], who noted that PEDOT:PSS has a large work function (about 5.0 eV) and conductivity, and it is transparent in the 250–280 nm wavelength range. This makes it suitable as a Schottky electrode for inorganic n-type wide-bandgap semiconductors like ZnO and Ga_2_O_3_. They then made a flame detector based on a PEDOT:PSS/Ga_2_O_3_ Schottky junction. The device has a structure of PEDOT:PSS/Ga_2_O_3_ semi-insulating layer/n-Ga_2_O_3_/In ohmic contact layer. Instead of epitaxial and vacuum processes, they used the floating zone method to grow single crystal Ga_2_O_3_ substrates, polished them, placed an In metal block on the substrate back, and annealed it in oxygen at 1100 °C. This fixed the In metal block and formed a semi-insulating layer and good ohmic contact. The PEDOT:PSS film was made by spin-coating a PEDOT:PSS aqueous solution with 5% dimethyl sulfoxide (DMSO), showing good conductivity and high transparency. The detector’s spectral response has a large R250 nm/R300 nm suppression ratio (1:5104) and an external quantum efficiency of 18% at 250 nm. The device’s flame-detection ability was tested under four conditions: (I) darkness; (II) fluorescent lamp illumination; (III) igniting a lighter near the device; (IV) extinguishing the lighter. Millivolt-level signals were used to assess the device’s response, as shown in Figure 7a. Results showed that without a visible-cut-off filter, the device could distinguish solar-blind light (1.5 nW/cm^2^) and flames under strong fluorescent light, proving the success of Ga_2_O_3_ detectors in flame detection and their potential for applications like furnace control and gas-flame detection.

**Figure 8:**
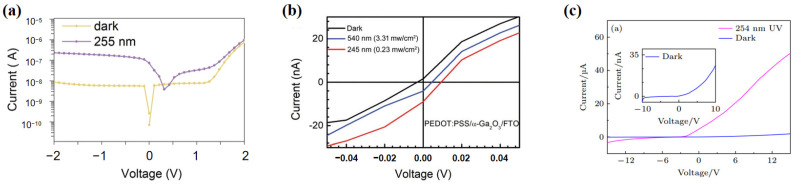
I–V characteristic curves of some PEDOT:PSS/Ga_2_O_3_ detectors under 0 V. (**a**) I–V characteristic curve of the PEDOT:PSS/Ga_2_O_3_/p-Si device in dark and under 255 nm illumination, reproduced with permission from Zhang D. et al. [85], Ultrahigh EQE (15%) Solar-Blind UV Photovoltaic Detector with Organic–Inorganic Heterojunction via Dual Built-In Fields Enhanced Photogenerated Carrier Separation Efficiency Mechanism; published by WILEY-V C H VERLAG GMBH (Weinheim, Germany), 2019. (**b**) I–V characteristic curve of the PEDOT:PSS/a-Ga_2_O_3_/FTO device under 245 nm and 540 nm illumination, reproduced with permission from Fan, M.-M. et al. [75], self-powered Solar-blind UV/visible Dual-band Photodetection based on a Solid-state PEDOT:PSS/α-Ga_2_O_3_ Nanorod Array/FTO Photodetector; published by ROYAL SOC CHEMISTRY (London, UK), 2021. (**c**) I–V curves of PEDOT:PSS/β-Ga_2_O_3_ devices under dark and 254 nm light illumination—inset shows the I–V curve of the device in dark, reproduced with permission from Zi-Qi, Y. et al. [74], performance of UV photodetector of mechanical exfoliation prepared PEDOT:PSS/β-Ga_2_O_3_ microsheet heterojunction; published by CHINESE PHYSICAL SOC (Beijing, China), 2024.

Ga_2_O_3_ inorganic heterojunction detectors often have lattice mismatch, which affects their external quantum efficiency and suppression ratio. Because Ga_2_O_3_’s crystal structure differs much from common substrates like SiC and sapphire, choosing a lattice-matched substrate or adding a buffer layer cannot reduce the interface state density. Zhang et al. [85] focused on PEDOT:PSS, which is less sensitive to surface defects. They proposed building a PEDOT:PSS/Ga_2_O_3_ heterojunction (top heterojunction) on the surface of epitaxially grown Ga_2_O_3_ films with distorted lattices. The mechanism is that under illumination, most photo-generated carriers in Ga_2_O_3_ are directly separated by the built-in electric field of the top junction, reducing the probability of carriers being trapped by interface states in the lower heterojunction (Ga_2_O_3_/substrate heterojunction). They spin-coated a PEDOT:PSS layer on the Ga_2_O_3_ film surface to make a PEDOT:PSS/Ga_2_O_3_/p-Si hybrid SB UV PD. Using the double built-in electric field of the heterojunction, the device’s EQE greatly increased, reaching 15% at zero bias, and the R255 nm/R405 nm suppression ratio was about 450, with an open-circuit voltage of about 0.57 V. Figure 8a illustrates the I–V characteristic curve of the device in dark and under 255 nm illumination. This offers important insights for making high-performance Ga_2_O_3_ UV PDs.

Similarly, Wang et al. [86] reported a high-performance self-powered solar-blind ultraviolet photodetector based on a PEDOT:PSS/β-Ga_2_O_3_ organic–inorganic p-n junction (Figure 7b). The device utilized highly crystalline β-Ga_2_O_3_ and the excellent transparent conductive polymer PEDOT:PSS, achieving an ultra-high responsivity of 2.6 A/W at a wavelength of 245 nm, and exhibiting a sharp cutoff wavelength at 255 nm. The solar-blind/ultraviolet rejection ratio (R_245nm_/R_280nm_) of the photodetector reached 10^3^, which is two orders of magnitude higher than that of previously reported Ga_2_O_3_-based SB PDs. In addition, the spectral response window of the device was only 17 nm, demonstrating excellent spectral selectivity. Notably, the device exhibited extremely low dark current (0.5 pA) under zero bias, a detection rate as high as 2.2 × 10^13^ Jones, and fast response times (rise time 0.34 ms, fall time 3 ms). This study provides new ideas for the development of high-responsivity, high-wavelength-selective, self-powered, solar-blind, ultraviolet photodetectors and showcases the great potential of organic–inorganic hybrid heterojunctions in the field of ultraviolet detection.

In addition to β-Ga_2_O_3_, detectors made from other Ga_2_O_3_ polymorphs are also of great concern. Fan et al. [75] first prepared an α-Ga_2_O_3_ nanorod array on an FTO substrate via a hydrothermal method and low-temperature annealing. Then, by spin-coating a PEDOT:PSS aqueous solution and annealing, they successfully fabricated a self-powered PEDOT:PSS/α-Ga_2_O_3_ nanorod array/FTO photodetector. For comparison, they also fabricated an Au/α-Ga_2_O_3_ nanorod array/FTO heterojunction using thermal evaporation. Notably, the strong built-in electric field at the PEDOT:PSS/α-Ga_2_O_3_ interface enabled the device to achieve the first dual-band DUV/visible photo-detection in a self-powered gallium oxide detector. It had a solar-blind UV main band with a peak response rate of about 1.43 mA W^−1^ at 245 nm and a visible photon sub-band with a peak response rate of about 0.07 mA W^−1^ at 540 nm, thus broadening the application prospects of self-powered gallium oxide detectors. Moreover, the peak response rate of this device in the solar-blind UV range was approximately 10⁴ times that of the control group’s α-Ga_2_O_3_ nanorod array/FTO PD (without a conductive polymer). It stood out among the popular self-powered α-Ga_2_O_3_ liquid-state photoelectrochemical PDs. Figure 8b illustrates the photoelectric properties of the device under 245 nm and 540 nm illumination. This study fabricated a high-performance solid-state gallium oxide SB UV/visible dual-band PD through a simple and low-cost process, offering high reference value.

In a recent report, Yi et al. [74] used mechanical exfoliation to obtain β-Ga_2_O_3_ single-crystal microsheets. They coated half of the microsheet with PEDOT:PSS solution, dried it, and then fabricated electrodes to form a heterojunction UV detector. By measuring the I–V characteristics of the device in the dark and under 254 nm UV illumination, it was found that the heterojunction exhibited good rectification and sensitivity to 254 nm UV light. The device could operate at zero external bias, with a response rate of 7.13 A/W and an external quantum efficiency as high as 3484%. The rise and fall times were 0.25 s and 0.20 s, respectively, indicating a significant improvement in self-powered performance compared to previous similar detectors. Figure 8c illustrates the I-V curves of the device under dark and 254 nm light illumination. Notably, after three months, the device’s sensitivity to 254 nm UV light showed no decay, demonstrating excellent temporal stability.

**Figure 9:**
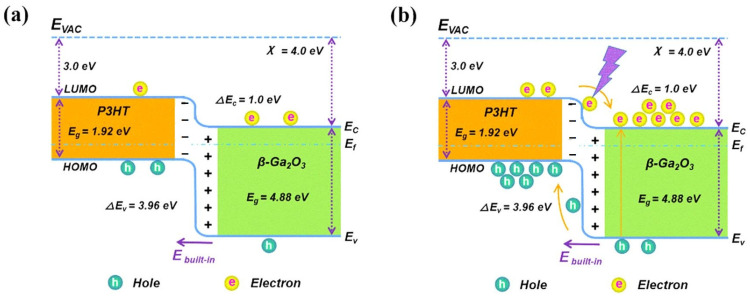
(**a**) Energy band diagram of the mixed P3HT/Ga_2_O_3_ heterojunction device in a dark environment. (**b**) Energy band diagram of the mixed P3HT/Ga_2_O_3_ heterojunction device under 254 nm irradiation, reproduced with permission from Qi, X. et al. [81], a self-powered deep-ultraviolet photodetector based on a hybrid organic-inorganic p-P3HT/n-Ga_2_O_3_ heterostructure; published by IOP Publishing Ltd., 2022.

Poly (3-hexylthiophene) (P3HT), a typical polythiophene derivative, exhibits p-type semiconductor characteristics with a high hole transport rate and good stability [87,88]. Its high solubility, due to alkyl side chains, lowers large-scale production costs and enhances solution crystallinity and morphology, boosting charge transport efficiency [89,90]. As a high-performance conductive polymer, P3HT is widely used in optoelectronic devices, including organic light-emitting diodes (OLEDs), photodetectors, and solar cells [88,91]. Qi et al. [81] used a spin-coating process to fabricate a planar-structured P3HT/β-Ga_2_O_3_ heterojunction self-powered UV photodetector. Thanks to the built-in electric field within the heterojunction, the device shows improved photoelectric performance with increased photocurrent and response speed, achieving an excellent response rate of 57.2 mA W^−1^ and a high detection rate of 1.47 × 10^17^ Jones. The open-circuit voltage was measured to be 0.26 V, indicating that it is an excellent self-powered device. The device demonstrates good stability and repeatability under different light intensities and voltages, attributed to the excellent spectral selectivity of gallium oxide, the high conductivity of P3HT, and the appropriate band alignment of the heterojunction (Figure 9). This offers new valuable application avenues for P3HT materials and Ga_2_O_3_ detectors.

### 5.2. Types of Polyaniline/Ga_2_O_3_-Based UV PDs

Polyaniline (PANI) is an important and promising conductive polymer (CP) and one of the most extensively studied. It is easy to synthesize, environmentally stable, and resistant to degradation. Its broad and tunable electrical conductivity makes it a low-cost and widely applicable composite material. PANI exists in multiple oxidation states, and its electrical conductivity varies with these forms, enabling conductivity tuning through oxidation state control [92]. PANI is typically prepared via chemical or electrochemical oxidative polymerization in acidic media [93]. Acid serves a dual role as both the polymerization medium and dopant, allowing doping and polymerization to occur in a similar manner during synthesis. Despite some drawbacks, such as limited solubility, lower conductivity compared to metallic conductors, poor mechanical properties, sensitivity to dopants, and potential environmental concerns [94], PANI’s exceptional protonic and redox doping capabilities make it a highly potential conductive polymer. By adjusting the doping level of PANI, various nano-composite materials with good electrical conductivity can be fabricated.

**Figure 10:**
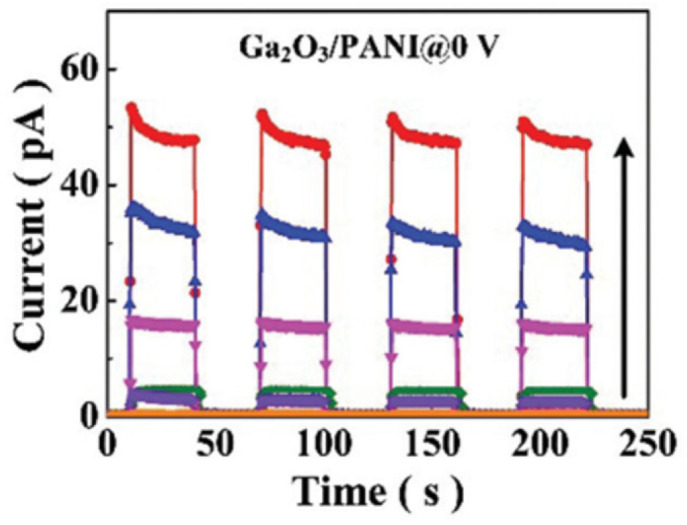
I–t characteristics of β-Ga_2_O_3_/PANI in the dark and under 250 nm, 260 nm, 270 nm, 280 nm, 320 nm, and 400 nm illumination at the voltage bias of zero. The curves from top to bottom were obtained respectively when the wavelengths of the incident light were 250, 260, 270, 280, 320, 400 and there was no light, reproduced with permission from Wang L. et al. [95], an ultrahigh responsivity self-powered solar-blind photodetector based on a centimeter-sized β-Ga_2_O_3_/polyaniline heterojunction.; published by ROYAL SOC CHEMISTRY, 2020.

The PANI/Ga_2_O_3_ heterojunction is also one of the most studied Ga_2_O_3_-based hybrid organic–inorganic heterojunctions. The weak interface interaction within the hybrid system effectively circumvents the generation of interface trap centers resulting from lattice mismatch. Additionally, the (-NH_2_)-^+^ ions in polyaniline stabilize the (O^2−^) anions, thereby passivating the oxygen vacancies in gallium oxide. This reduces the concentration of oxygen vacancies and mitigates persistent photoconductivity. Furthermore, the polyaniline layer can serve a dual function of band-pass transmission and hole-extended transmission in solar-blind ultraviolet detectors. It can not only suppress the photoresponse of visible light but also improve the collection efficiency of photogenerated carriers. As the internal photoemission effect is negligible, it also improves the rejection ratio. Wang et al. [95] synthesized centimeter-level β-Ga_2_O_3_ microwires with high crystal quality through CVD, and then grew highly doped PANI on the surface of the microwires via in situ polymerization in an acidic aqueous solution, demonstrating a self-powered photodetector based on a β-Ga_2_O_3_ microwire/PANI hybrid heterojunction. Benefiting from the excellent photoelectric properties of Ga_2_O_3_ and the strong hole-transport capability of PANI, the device exhibited ultra-high response performance. Figure 10 demonstrates the I-t characteristics of the device in the dark and under 250 nm, 260 nm, 270 nm, 280 nm, 320 nm, and 400 nm illumination at the voltage bias of zero. At the peak (246 nm), the device’s responsivity was 21 mA/W, and the cutoff edge at 272 nm was just in the solar-blind ultraviolet spectrum region. The rise time was measured to be about 0.34 ms, and the decay time was about 8.14 ms. Under a bias of 0.1 V, the device had an extremely low dark current of only 0.3 pA and a high detection rate of up to 1.5 × 10^11^ cm Hz^−12^ W^−1^, which can be used for weak signal detection in the solar-blind region and other fields.

**Figure 11:**
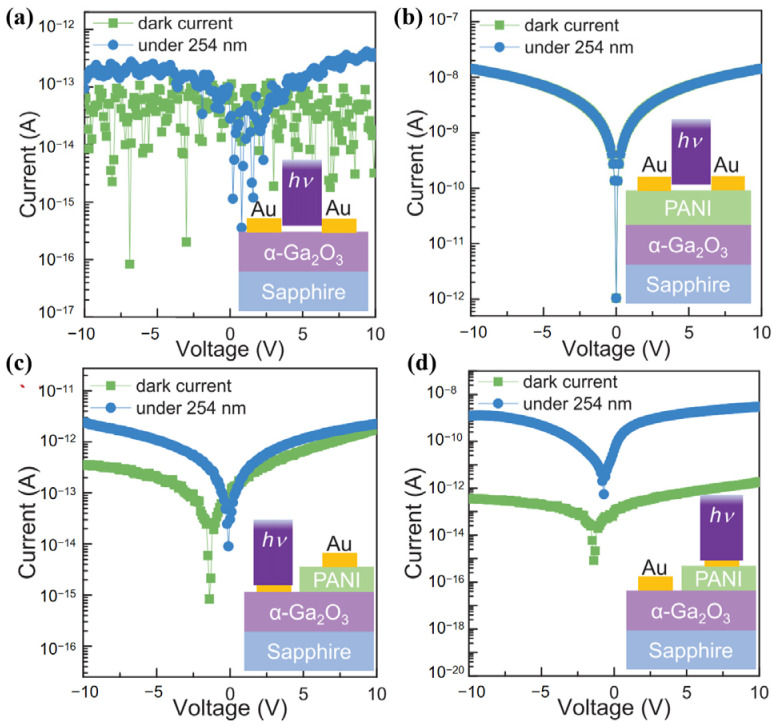
The current–voltage (I–V) characteristics of diodes under the dark condition and 254 nm DUV irradiation with four testing configurations shown in the inset: (**a**) Au/α-Ga_2_O_3_/Au, (**b**) Au/PANI/Au, (**c**) Au/α-Ga_2_O_3_/PANI/Au illuminated upon Au/α-Ga_2_O_3_ contact area and (**d**) Au/α-Ga_2_O_3_/PANI/Au illuminated upon α-Ga_2_O_3_/PANI area, reproduced with permission from Sun X.Y. et al. [66], a self-powered solar-blind photodetector based on polyaniline/α-Ga_2_O_3_ p--n heterojunction; published by AIP Publishing, 2021.

Sun et al. [66] tried to form a heterojunction with other phases of gallium oxide and PANI to make a UV PD and enhance its photoelectric properties. They deposited an α-Ga_2_O_3_ epitaxial film on a sapphire substrate using a homemade mist-CVD system and grew a PANI film on it via hydrothermal self-assembly, creating a self-powered SB UV photodetector based on a polyani/α-Ga_2_O_3_ hybrid heterojunction. Thanks to the interfacial built-in field, the device shows great self-powered detection and fast response, with a zero-bias peak response of 8.2 mA/W, a suppression ratio (R220 nm/R400 nm) of 2.97 × 10^4^, and a response decay time (sdec) of 176 μs. When a 5 V bias is applied, the dark current stays ultra-low at 0.21 pA, while the suppression ratio and fall time improve to 7.13 × 10^4^ and 153 μs, respectively. The corresponding external quantum efficiency is 38.4%, and the detectivity is 6.63 × 10^13^ Jones. Figure 11 illustrates the I–V characteristics of diodes under the dark condition and 254 nm DUV irradiation. This work, with its low-temperature process and excellent properties, offers new design solutions for high-performance gallium oxide detectors and shows application potential in deep-UV photonics and power electronics.

### 5.3. Types of Polycarbazoles/Ga_2_O_3_-Based UV PDs

In addition to polythiophene derivatives and polyaniline, researchers are also focusing on finding more promising conductive polymers. Among them, polycarbazole derivatives such as PCDTBT and PVK have shown better performance and have received more attention.

**Figure 12:**
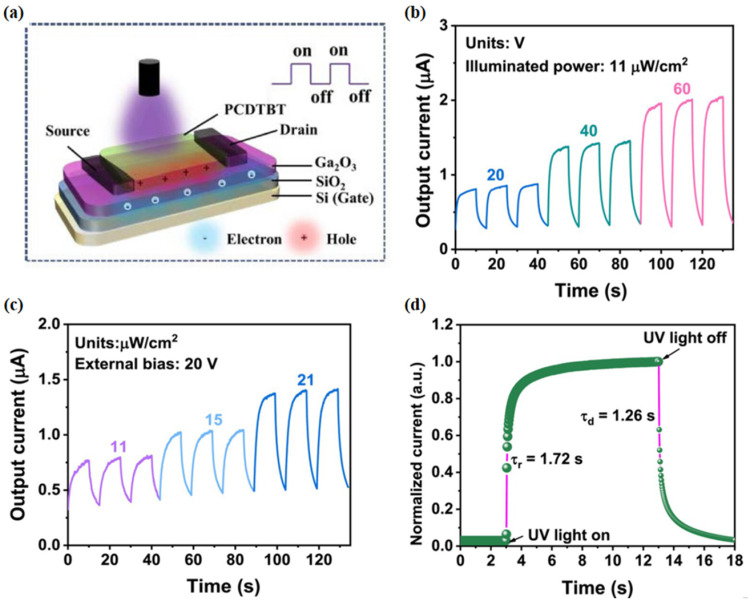
(**a**) Schematic diagram of a photodetector based on the PCDTBT/a-Ga_2_O_3_ heterojunction under periodic UV pulse irradiation. (**b**) Time response of the PCDTBT/a-Ga_2_O_3_ phototransistor under 255 nm UV light at different voltages (20, 40, and 60 V). (**c**) Time response of the PCDTBT/a-Ga_2_O_3_ phototransistor under 255 nm UV light at different light intensities (11, 15, and 21 μW cm^−2^). (**d**) Normalized time response spectrum of the PCDTBT/a-Ga_2_O_3_ phototransistor, reproduced with permission from Wang Y. et al. [67], multifunctional solar-blind ultraviolet photodetectors based on p-PCDTBT/n-Ga_2_O_3_ heterojunction with high photoresponse; published by WILEY, 2024.

Poly [N-9′-heptadecanyl-2,7-carbazole-alt-5,5-(4′,7′-di-2-thienyl-2′,1′,3′-benzothiadiazole)] (PCDTBT) is a conjugated poly (2,7-carbazole) derivative with a high glass transition temperature, good solubility, relatively high molecular weight, and air stability. Due to the ultrafast photo-induced charge transfer process within the system, PCDTBT has high fluorescence efficiency [96]. In addition, PCDTBT has a narrow bandgap (Eg = 1.8 eV) and good carrier mobility and has been widely used as a transport layer in organic thin film transistors and organic solar cells [97,98]. Wang et al. [67] innovatively used this conductive polymer and amorphous gallium oxide (a-Ga_2_O_3_) to successfully manufacture a multifunctional SB UV PD based on a p-PCDTBT/n-Ga_2_O_3_ hybrid heterojunction. The device can work in a photo-transistor mode coupled with self-powered, and the depletion effect of PCDTBT on a-Ga_2_O_3_ can increase the transistor threshold voltage and reduce the device’s dark current to 0.48 pA. After modulation by the PCDTBT layer, the device showed a uniform surface, excellent photoelectric properties, and excellent stability. Under a weak light intensity of 11 μW/cm^–2^, the responsivity, photo-detection rate, and external quantum efficiency were increased to 187 A/W, 1.3 × 10^1^⁶ Jones, and 9.1 × 10^4^%, respectively. Figure 12 shows the method and results for testing the time-performance of the device, which indicates that the device has good time-response characteristics. This study provides a reference pathway for the wide application of photodetectors based on amorphous Ga_2_O_3_.

**Figure 13:**
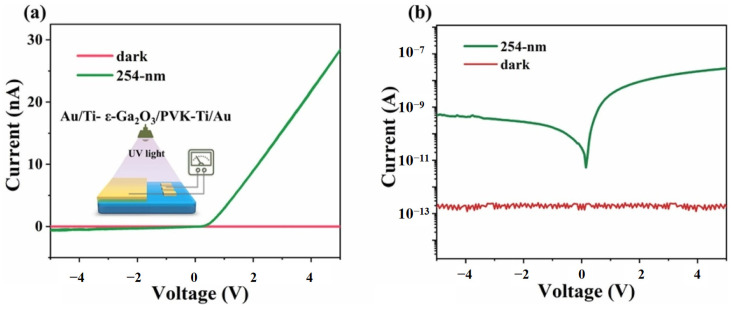
(**a**,**b**) I–V characteristics of Au/Ti-PVK/(ε-Ga_2_O_3_)-Ti/Au hybrid device, reproduced with permission from Dai J. et al. [76], fabrication of a poly(N-vinyl carbazole)/ε-Ga_2_O_3_ organic–inorganic heterojunction diode for solar-blind sensing applications; published by IOP Publishing Ltd., 2021.

Another widely studied conductive polymer is poly (N-vinyl carbazole) (PVK), a hole-transporting organic semiconductor polymer with a bandgap of about 3.6 eV [99]. PVK has good thermal and chemical stability, high photoconductivity, and transparency, making it an efficient hole-transporting layer with important applications in OLEDs and photodetectors (PDs). Dai and his colleagues [76] used PVK and another little-developed gallium oxide polycrystal—ε-Ga_2_O_3_—to fabricate a photodetector based on a PVK/Ga_2_O_3_ heterojunction. Previous studies have shown that ε-Ga_2_O_3_ has a wide bandgap and extremely high dark resistivity, and epitaxial layers can be directly deposited on the c-plane sapphire substrate to manufacture ε-Ga_2_O_3_ thin films. This simple and cost-effective photoresistor has good performance, so it is possible to design and manufacture solar-blind ultraviolet photodetectors. Under 254 nm ultraviolet light irradiation, the organic-inorganic hybrid photodiode exhibited obvious rectification characteristics at ±2 V. At 5 V, it had a rise time of 0.52 s and a decay time of 0.11 s, indicating good response speed of the device. The V_oc_ showed a constant value of approximately 0.18 V because it is only related to the energy-band alignment of the interface. The I–V characteristics diagram of the device are shown in Figure 13. The use of new conductive polymer materials and new polycrystalline gallium oxide has provided more pathways for the in-depth application of gallium oxide SB UV PDs.

Here is a summary of the photoresponse performance of the main types of thin-film Ga_2_O_3_/conductive polymers hybrid heterojunction ultraviolet photodetectors in Table 1:

## 6. Summary and Outlook

This paper comprehensively reviews the latest progress in solar-blind UV photodetectors based on Ga_2_O_3_/conductive polymer (CPs) heterojunctions. A comparison shows that polythiophene has a high electrical conductivity and a flexibly tuneable polymer bandgap for measuring different wavelengths. It is suitable for highly conductive and tuneable devices, and it is currently the most intensively studied. However, the sulfur atoms in polythiophene derivatives are prone to oxidation, resulting in poor environmental stability. Polyaniline allows control of conductivity through its oxidation state and can achieve a broadband spectral response upon doping. It also has simple device-fabrication processes and a low cost, and it is widely studied. Nevertheless, it has low solubility and a strong dependence on acidic doping. Polycarbazole has high thermal stability and hole-transport advantages, and devices made from it have high luminous efficiency, making it suitable for environments with thermal stability requirements. However, polycarbazole derivatives have high HOMO energy levels and low LUMO energy levels, causing an energy level mismatch and weak electron-transport properties. At present, research on polycarbazole is relatively limited.

The response time of Ga_2_O_3_-based photodetectors is significantly influenced by the type of conductive polymer. The polymer’s molecular structure and degree of conjugation affect its conductivity and band regulation ability. The energy level matching determines the separation and transport efficiency of photogenerated carriers. Differences in carrier mobility across polymers directly affect carrier transport speed, leading to significant variations in how different polymers enhance the response time of Ga_2_O_3_-based photodetectors. The mechanisms by which different polymers affect detector response speed also vary. Polythiophene has a rigid, planar molecular structure. Its ordered molecular arrangement optimizes interfacial properties, and sulfur atoms can fill oxygen vacancies to boost carrier transport speed. Polyaniline alters energy level matching via doping, influencing the separation and transport efficiency of photogenerated carriers. Polycarbazole, with its low transport energy barriers and high mobility, serves as a high-quality hole transport channel in heterojunctions. This accelerates carrier transport and improves response speed.

Thanks to the conductive polymer’s unique backbone with lots of conjugated π electrons, which brings high conductivity, easy processing, low cost, flexibility, and stability, the Ga_2_O_3_/conductive polymer heterojunction detector is better for carrier separation, improving photoelectric performance, and cutting manufacturing costs. It also enhances device flexibility and reduces lattice mismatch at the heterojunction interface, greatly boosting the suppression ratio and external quantum efficiency, and is widely used. In recent years, it has gained more attention and research.

However, research on Ga_2_O_3_/CPs heterojunction detectors still faces many challenges. First, in terms of photoelectric response properties, although some devices have achieved millisecond-level or faster response speeds, they still cannot meet the requirements of high-speed detection and have room for improvement. The dark current of the device is also of concern. Even under zero bias or reverse bias, some devices still have a large leakage current, which may be caused by defects at the pn junction or crystal interface. In addition, as the light intensity increases, the responsivity, detectivity, and external quantum efficiency of some devices show a downward trend. This is due to the self-heating effect of the heterojunction, which increases the probability of electron–hole recombination. Although most performance parameters are excellent, some device performances are still limited. For example, defects in the gallium oxide film may affect the self-powered performance of the device, and relatively narrow-bandgap semiconductors may affect the device’s cut-off wavelength and suppression ratio, leaving room for improvement in the device’s overall performance in the solar-blind region, among others. Fortunately, researchers are exploring feasible strategies to solve these problems. Using innovative processes, optimizing polymer doping concentrations, and employing band engineering and interface engineering, etc., are all helpful in improving the situation.

In conclusion, continuously optimizing the comprehensive performance of Ga_2_O_3_ heterojunction-based UV PDs and actively exploring their application fields have become the mainstream of research. With the deepening of research, Ga_2_O_3_ heterojunction detectors will be able to leverage their strengths and find applications in more fields.

## Figures and Tables

**Table 1:** Comparison of structures and performances of Ga_2_O_3_-based SB UV PDs.

Structures	Wavelength (nm)	R (A/W)	D* (Jones)	t_rise_/t_decay_ (s/s)	Ref.
PEDOT:PSS/Ga_2_O_3_	255	2.9 × 10^–2^	-	0.06/0.088	[85]
P3HT/Ga_2_O_3_	254	57.2 m	1.47 × 10^17^	0.16/0.01	[81]
PANI/Ga_2_O_3_	220	6.76 × 10^–2^	6.63 × 10^13^	0.36 m/1.76 m	[66]
PCDTBT/Ga_2_O_3_	255	187	1.3 × 10^16^	1.72/1.26	[67]
PVK/Ga_2_O_3_	254	-	-	0.52/0.11	[76]
GaN/Sn:Ga_2_O_3_	254	3.05	1.69 × 10^13^	-/18 m	[17]
NiO/Ga_2_O_3_	254	5.7 × 10^–2^	5.45 × 10^9^	0.34/3.65	[22]

## Data Availability

No new data were created or analyzed in this study. Data sharing is not applicable to this article.
