# Correction: Xiao et al. Self-Powered Ultraviolet Photodetectors Based on Conductive Polymers/Ga_2_O_3_ Heterojunctions: A Review. *Polymers* 2025, *17*, 1384

**DOI:** 10.3390/polym17192642

**Published:** 2025-09-30

**Authors:** Zerui Xiao, Haoyan Chen, Honglong Ning, Dongxiang Luo, Xuecong Fang, Muyun Li, Guoping Su, Han He, Rihui Yao, Junbiao Peng

**Affiliations:** 1 State Key Laboratory of Luminescent Materials and Devices, Guangdong Basic Research Center of Excellence for Energy and Information Polymer Materials, South China University of Technology, Guangzhou 510640, China; 202230274330@mail.scut.edu.cn (Z.X.); 202420119664@mail.scut.edu.cn (H.C.); 202421021983@mail.scut.edu.cn (X.F.); 202111084406@mail.edu.scut.cn (M.L.); 201730321254@mail.scut.edu.cn (G.S.); 202410184224@mail.scut.edu.cn (H.H.); psjbpeng@scut.edu.cn (J.P.); 2 The International School of Microelectronics, Dongguan University of Technology, Dongguan 523808, China; 3 Huangpu Hydrogen Innovation Center/Guangzhou Key Laboratory for Clean Energy and Materials, School of Chemistry and Chemical Engineering, Guangzhou University, Guangzhou 510006, China; luodx@gzhu.edu.cn

## Missing Citation

In the original publication [1], reference “Mattei, F.; Vurro, D.; Spoltore, D.; Pavesi, M.; Kalvani, P.R.; Pasini, S.; Foti, G.; D’Angelo, P.; Bosio, A.; Baraldi, A.; et al. Planar Hybrid UV-C Photodetectors Based on Aerosol-Jet Printed PEDOT:PSS on Different Ga_2_O_3_ Thin Films. *Mater. Today Phys.* **2025**, *51*, 101663” was not cited. The citation has now been inserted in Figure 1 of Section 1. Introduction and should read: Figure 1. Outline of conductive polymers/Ga_2_O_3_ self-powered ultraviolet photodetectors, reproduced from [34].

With this correction, the order of some references has been adjusted accordingly. The authors state that the scientific conclusions are unaffected. These corrections were approved by the Academic Editor. The original publication has also been updated.
